# AI-driven body composition monitoring and its prognostic role in mCRPC undergoing lutetium-177 PSMA radioligand therapy: insights from a retrospective single-center analysis

**DOI:** 10.1186/s13550-025-01312-9

**Published:** 2025-08-28

**Authors:** Tristan Ruhwedel, Julian Rogasch, Markus Galler, Imke Schatka, Christoph Wetz, Christian Furth, Nadine Biernath, Maria De Santis, Seyd Shnayien, Johannes Kolck, Dominik Geisel, Holger Amthauer, Nick Lasse Beetz

**Affiliations:** 1 Department of Nuclear Medicine, Charité – Universitätsmedizin Berlin, Corporate Member of Freie Universität Berlin and Humboldt-Universität zu Berlin, Augustenburger Platz 1, 13353 Berlin, Germany; 2 Department of Radiology, Charité – Universitätsmedizin Berlin, Corporate Member of Freie Universität Berlin and Humboldt-Universität zu Berlin, Augustenburger Platz 1, 13353 Berlin, Germany; 3 Berlin Institute of Health at Charité – Universitätsmedizin Berlin, Charitéplatz 1, 10117 Berlin, Germany; 4 Department of Urology, Charité – Universitätsmedizin Berlin, Corporate Member of Freie Universität Berlin and Humboldt-Universität zu Berlin, Charitéplatz 1, 10117 Berlin, Germany; 5 Department of Urology, Medical University of Vienna, Vienna, Austria

**Keywords:** Prostate cancer, Radioligand therapy, Artificial intelligence, Body composition, PET/CT, Prognosis, Visceral adipose tissue, Overall survival

## Abstract

**Background:**

Body composition (BC) analysis is performed to quantify the relative amounts of different body tissues as a measure of physical fitness and tumor cachexia. We hypothesized that relative changes in body composition (BC) parameters, assessed by an artificial intelligence–based, PACS-integrated software, between baseline imaging before the start of radioligand therapy (RLT) and interim staging after two RLT cycles could predict overall survival (OS) in patients with metastatic castration-resistant prostate cancer.

**Methods:**

We conducted a single-center, retrospective analysis of 92 patients with mCRPC undergoing [^177^Lu]Lu-PSMA RLT between September 2015 and December 2023. All patients had [^68^ Ga]Ga-PSMA-11 PET/CT at baseline (≤ 6 weeks before the first RLT cycle) and at interim staging (6–8 weeks after the second RLT cycle) allowing for longitudinal BC assessment.

**Results:**

During follow-up, 78 patients (85%) died. Median OS was 16.3 months. Median follow-up time in survivors was 25.6 months. The 1 year mortality rate was 32.6% (95%CI 23.0–42.2%) and the 5 year mortality rate was 92.9% (95%CI 85.8–100.0%). In multivariable regression, relative change in visceral adipose tissue (VAT) (HR: 0.26; *p* = 0.006), previous chemotherapy of any type (HR: 2.4; *p* = 0.003), the presence of liver metastases (HR: 2.4; *p* = 0.018) and a higher baseline De Ritis ratio (HR: 1.4; *p* < 0.001) remained independent predictors of OS. Patients with a higher decrease in VAT (< −20%) had a median OS of 10.2 months versus 18.5 months in patients with a lower VAT decrease or VAT increase (≥ −20%) (log-rank test: *p* = 0.008). In a separate Cox model, the change in VAT predicted OS (*p* = 0.005) independent of the best PSA response after 1–2 RLT cycles (*p* = 0.09), and there was no interaction between the two (*p* = 0.09).

**Conclusions:**

PACS-Integrated, AI-based BC monitoring detects relative changes in the VAT, Which was an independent predictor of shorter OS in our population of patients undergoing RLT

## Background

Prostate cancer remains one of the most frequently diagnosed cancers in men worldwide and is the fifth most common cancer-related cause of death in men [1]. Metastatic castration-resistant prostate cancer (mCRPC) is considered the incurable terminal stage and is associated with a limited overall survival (OS) of approximately 1–2 years from the time of diagnosis [2]. Despite numerous new therapeutic approaches, treatment of these patients remains challenging.

In addition to newer antihormonal drugs, taxane chemotherapy or PARP inhibitors for patients with BRCA or HRR alterated tumors, radioligand therapy (RLT) with [^177^Lu]Lu-labeled prostate-specific membrane antigen ligands has been approved for patients who have failed standard therapy. The prospective randomized controlled phase III VISION trial demonstrated a significantly prolonged OS of 15.3 months in patients who underwent RLT compared to 11.3 months in those who received standard therapy [3]. Furthermore, the prospective phase II TheraP trial showed a significantly better prostate-specific antigen response compared with cabazitaxel therapy [4]. Of note, however, OS showed no significant difference [5], and not all patients benefit from RLT. A recent meta-analysis found that one in four patients undergoing RLT experienced primary progression [6].

Several prognostic factors, including various clinical, laboratory, and imaging parameters, have been identified [7, 8]. Furthermore, imaging parameters such as the maximum or mean standardized uptake value (SUVmax or SUVmean), total-lesion PSMA (TLP) or the molecular-tumor volume (MTV) have also become the focus of research because, in a theranostic setting, the treatment response of patients undergoing RLT is often closely monitored using PSMA-positron emission tomography/computed tomography (PET/CT) [9, 10]. Gafita et al. proposed a nomogram for predicting OS after RLT based on two prospective phase II trials that was externally validated. The nomogram included time since diagnosis, chemotherapy status, pretherapeutic hemoglobin level, total number of metastases, SUVmean as well as bone or liver involvement showed good prognostic stratification capabilities [11].

In the context of prostate cancer and RLT, understanding body composition (BC) becomes important as patients suffering from conditions such as sarcopenia, cachexia, or obesity are at an increased risk for longer hospital stays, perioperative complications, and reduced overall survival [12–15]. Proper identification of these at-risk patients is essential for improving treatment outcomes. Unlike the traditional body mass index (BMI), artificial intelligence (AI)-based analysis of BC, with parameters that can be determined in a CT scan on a single slice at the level of L3, offers a more detailed assessment by differentiating the relative proportions of various tissues. This includes evaluating muscle and adipose tissue parameters such as skeletal muscle index (SMI), psoas muscle index (PMI), visceral adipose tissue (VAT), and subcutaneous adipose tissue (SAT). Such metabolic information from individualized BC analysis may identify frail patients undergoing RLT.

Most recently, pretherapeutic BC parameters have been identified as prognostic factors in various oncological diseases, hereditary connective tissue diseases, and cardiovascular diseases [16–19]. It has also been shown that changes in these parameters can indicate poorer prognosis in various diseases [20]. Furthermore, a recently published study has failed to demonstrate any prognostic value of pretherapeutic BC parameters in patients undergoing RLT [21]. However, despite the missing prognostic value of pretherapeutic BC parameters, tracking their changes during therapy could potentially reveal insights into patient response and treatment efficacy that static measurements might miss. We therefore conducted a retrospective analysis to investigate intratherapeutic changes in BC parameters and their prognostic value regarding OS in patients with mCRPC undergoing RLT.

## Methods

### Patient population

We conducted a single-center, retrospective analysis of 92 patients with mCRPC undergoing RLT between September 2015 and December 2023. Thirty-three (36%) of these patients had been included in an earlier manuscript that focused exclusively on baseline laboratory predictors, while no body-composition or longitudinal imaging data were analysed in that study [8]. The following inclusion criteria were defined: (1) histologically and clinically confirmed diagnosis of mCRPC, (2) positivity of PSMA expression in functional imaging (PSMA-PET/CT) prior to RLT (3) initial staging with [^68^ Ga]Ga-PSMA-11-PET/CT as baseline imaging within 6 weeks before the first cycle of RLT, (4) at least two cycles of RLT (5) interim staging [^68^ Ga]Ga-PSMA-11-PET/CT within 6 to 8 weeks after the second cycle of RLT, and (6) follow-up ≥ 12 months from the day of administration of the first cycle of RLT, if not previously deceased. [^18^F]FDG-PET/CT was usually not performed prior to RLT.

### Image acquisition

PET/CT examinations were performed in our center with either a Philips Gemini TF 16 scanner with time-of-flight capability and a 16-row CT scanner [22] or a GE Discovery MI scanner with silicon photomultipliers and time-of-flight capability and a 64-row CT scanner [23]. All patients underwent CT with administration of an intravenous bolus injection of 80–120 ml iodinated contrast medium with a flow rate of 2–3 ml/s, followed by a venous-phase helical scan from skull base to mid-thigh (80 s delay).

### Radioligand therapy and evaluation

Patients underwent RLT with a median of 3 cycles (range: 2–8 cycles) and a scheduled dose of either 200 mCi (7.4 GBq) [^177^Lu]Lu-PSMA-617 or 200 mCi (7.4 GBq) [^177^Lu]Lu-PSMA-I&T. Additionally, patients underwent baseline staging with [^68^ Ga]Ga-PSMA-11-PET/CT within 6 weeks before the start of RLT and another interim staging with [^68^ Ga]Ga-PSMA-11-PET/CT 6–8 weeks after the second cycle of RLT, which was repeated every two cycles. The disease progression was diagnosed by an interdisciplinary tumor board with a PSMA-PET/CT. In patients with progressive disease, RLT was discontinued and no further RLT cycles were administered. OS was defined as the period from the administration of the first cycle of RLT to the date of death from any cause. For patients who were alive at the time of analysis, OS was censored on the date they were last known to be alive. The formal study cut-off date was 1 July 2024. In patients showing a good response after 6 cycles of RLT, another 2 cycles could be applied as part of an individualized concept, if no other treatment option was available.

### Body composition

All PET/CT imaging datasets were acquired at our Department of Nuclear Medicine. BC analysis was performed using an AI-based image segmentation tool that has already been validated in several studies [16, 18, 20, 24]. BC-analysis was performed using the 5-mm-thick contrast-enhanced CT images from the PET/CT scans, which were obtained as described above for the baseline and interim staging. We used a picture archiving and communications system (PACS)-integrated AI-based software tool (Visage version 7.1, Visage Imaging GmbH, Berlin, Germany), which is based on a convolutional neural network [24]. The network consists of nine blocks: four upsampling blocks, four downsampling blocks, and one in between. The initial training data consisted of 200 axial CT images of the third lumbar vertebra (L3) level, which were acquired at different sites using various CT protocols. Skeletal muscle, psoas muscle, and visceral and subcutaneous fat tissue were automatically separated. Each tissue class was coded with a different color. Automatic segmentation was checked by an experienced radiologist. AI-based image segmentation was manually corrected in few cases to avoid false area calculation, for example, when hypodense stool in the intestine was misinterpreted as body fat. For each tissue class, the software tool automatically calculated the area in square centimeters [cm^2^] and density in Hounsfield units [24] The SMI was calculated using the following formula: skeletal muscle area including the psoas muscle [cm^2^]/(body surface area [m])^2^. Furthermore, the PMI was calculated using the following formula: psoas muscle area [cm^2^]/(body surface area [m])^2^. For internal and external validation of the AI software tool, its performance has already been compared with that of an established semiautomatic segmentation tool in terms of speed and accuracy of tissue area calculation.

**Fig. 1:**
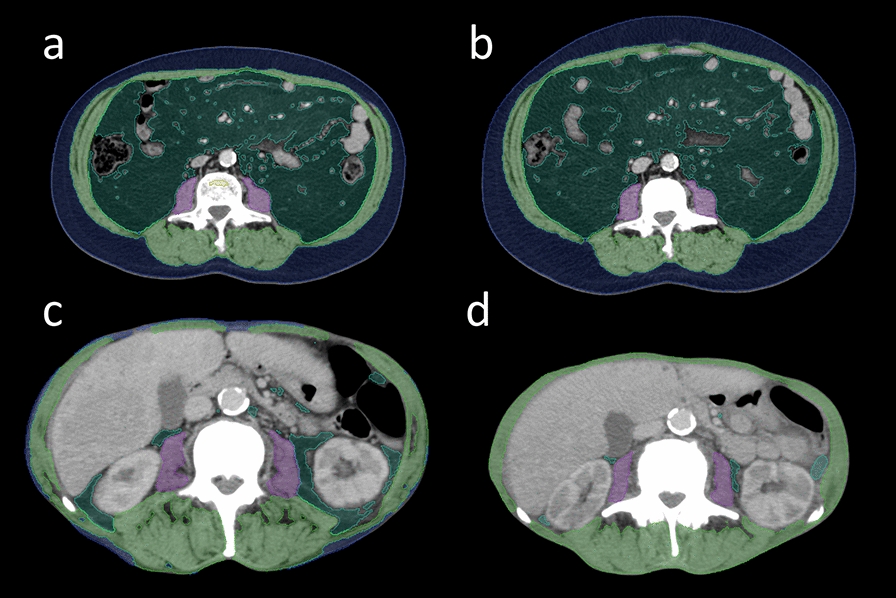
Representative patients illustrating visceral adipose tissue gain versus loss. Visceral adipose tissue (VAT) is highlighted in dark green. The figure shows two representative patients showing gain of VAT (**a/b**) and loss of VAT (**c/d**) between baseline PSMA-PET/CT before starting radioligand therapy (RLT) (a/c) and interim staging performed 6 to 8 weeks after the second cycle of RLT (b/d). The patient in the upper row remained alive for > 60 months before being lost to follow-up, whereas the patient in the lower row showed an overall survival of 7.3 months. Blue = subcutaneous adipose tissue; Light green = skeletal muscle; Purple = psoas muscle

Relative changes in BC parameters were calculated by subtracting the baseline staging value from the interim staging value and then dividing this value by the baseline value. Figure 1 illustrates a patient showing loss of VAT.

### Statistical analysis

Statistical analysis was performed using SPSS version 26 (IBM, Chicago, IL, USA) and R 4.3.2 (Foundation for Statistical Computing, Vienna, Austria, 2023; http://www.R-project.org (accessed on 05 November 2023)). Significance was assumed at α = 0.05. Descriptive values were expressed as median, interquartile range (IQR) and range. Univariable Cox proportional hazards regression for estimating OS was performed including clinical parameters (age, height, initial Gleason score, therapy before RLT (radical prostatectomy, androgen deprivation therapy (ADT), abiraterone, enzalutamide, radiation therapy, radium-223 (Ra-223), chemotherapy (yes vs. no))), localization of metastases (lymph node, bone, liver, lungs, brain, soft tissue, adrenal gland), absolute baseline values as well as relative changes in body composition parameters (BMI, PMI, SMI, VAT and SAT), and pretherapeutic laboratory parameters (prostate-specific antigen (PSA), creatinine, urea, uric acid, De Ritis ratio, leukocytes, thrombocytes, hemoglobin). The hazard ratio (HR) and the 95% confidence interval (CI) of the HR were determined for each parameter. All variables with *p* ≤ 0.1 in univariable Cox regression were also candidates for stepwise inclusion into multivariable Cox regression (criterion: likelihood ratio). The proportional hazard assumption was tested using the goodness-of-fit test and fulfilled by each variable. The best PSA response was defined as the highest decrease in PSA after either 1 or 2 cycles of RLT, relative to the pretherapeutic baseline. In a separate multivariable Cox regression analysis, we included the relative change in VAT and the best PSA response after 1 to 2 cycles of RLT as well as their interaction term (relative change in VAT * best PSA response) to investigate both the individual effects of each of these variables and their combined effect on OS after therapy. For the relative change in VAT, a cutoff was then formed using the value that achieved the minimum *p*-value in the log-rank test as determined with the Charité cutoff finder [25]. Descriptive variables were compared between groups with high decrease vs. low decrease/increase in VAT using the two-tailed Wilcoxon rank-sum test (continuous variables) or Fisher’s exact test (categorical variables). Absolute values of BC parameters were compared between baseline staging and interim staging using the Wilcoxon signed-rank test. The Kaplan–Meier method was used to estimate survival rates and median OS.

## Results

### Patient characteristics

Overall, 92 patients were included in this analysis. Eighty-three patients (90.2%) underwent at least one mCRPC-directed treatment (median 3; range 0–5) before their first cycle of RLT. In detail, 68 (73.9%) patients received abiraterone, 57 (62.0%) enzalutamide, and 6 (6.5%) radium-223 (Ra-223); 66 (71.7%) underwent chemotherapy, with 63 (68.5%) receiving docetaxel and 38 (41.3%) cabazitaxel. Sites of metastatic disease were distributed as follows: 86 (93.5%) patients suffered from bone metastases, 77 (83.7%) from lymph node metastases, 15 (16.3%) from pulmonary metastases, 10 (10.9%) from liver metastases, 9 (9.8%) from soft tissue metastases, and 2 (2.2%) from adrenal gland metastases. No patient had brain metastases. Patient characteristics are presented in Table 1.

**Table 1:** Patient characteristics

Variable	n (%) or Median (Range)			*p*
	Total study population	Relative change in VAT < −20%	Relative change in VAT ≥ −20%	
Number of patients	92	26	66	
Age (in years)	69 (50–87)	69 (52–81)	70 (50–87)	0.38
Height (in m)	1.76 (1.65–1.92)	1.76 (1.67–1.84)	1.76 (1.65–1.92)	0.68
Weight (in kg)	81 (47–125)	83 (47–105)	81 (59–125)	0.54
Initial Gleason score	9 (4–10)	9 (5–10)	9 (4–10)	0.77
Number of total RLT cycles	3 (2–8)	3 (2–6)	4 (2–8)	0.15
*Sites of metastases*				
Lymph node	77 (84%)	20 (77%)	57 (86%)	0.35
Bone	86 (94%)	24 (92%)	62 (94%)	1.0
Liver	10 (11%)	6 (23%)	4 (6%)	**0.028**
Lungs	15 (16%)	3 (12%)	12 (18%)	0.54
Soft tissue	9 (10%)	2 (8%)	7 (11%)	1.0
Adrenal gland	2 (2%)	1 (4%)	1 (2%)	0.49
*Pretreatment before RLT*				
Radical prostatectomy	47 (51%)	13 (50%)	34 (52%)	1.0
Androgen deprivation therapy	90 (98%)	25 (96%)	65 (99%)	0.49
Abiraterone	68 (74%)	21 (81%)	47 (71%)	0.44
Enzalutamide	57 (62%)	11 (42%)	46 (70%)	**0.018**
Radiation therapy	60 (65%)	19 (73%)	41 (62%)	0.47
Ra-223 therapy	6 (7%)	4 (15%)	2 (3%)	0.051
Chemotherapy of any type	66 (72%)	19 (73%)	47 (71%)	1.0
*Pretherapeutic laboratory values*				
PSA [ng/ml]	87 (0.01–10,000)	102 (3.7–10,000)	84 (0.01–1438)	0.24
Creatinine [mg/dl]	0.89 (0.5–1.7)	0.89 (0.5–1.7)	0.89 (0.5–1.5)	0.62
Urea [mg/dl]	35 (12–84)	33 (12–63)	36 (16–84)	0.66
Uric acid [mg/dl]	5.1 (2.0–9.8)	5.2 (2.0–7.3)	5.1 (2.8–9.8)	0.97
Leukocytes [/nl]	6.3 (2.4–13.1)	5.4 (2.4–13.1)	6.4 (3.0–10.3)	0.27
Thrombocytes [/nl]	251 (54–555)	256 (56–555)	250 (54–506)	0.71
Hemoglobin [g/dl]	12.2 (6.4–15.6)	10.8 (6.4–14.7)	12.4 (7.5–15.6)	**0.013**
De Ritis ratio	1.6 (0.6–7.3)	1.6 (0.8–7.3)	1.6 (0.6–5.6)	0.14

Patient characteristics are provided for the total study population and for subgroups with a high decrease in VAT (< −20%) and low decrease or increase in VAT (≥ −20%). The two subgroups were compared using either a two-tailed Fisher’s exact test or Wilcoxon rank-sum test. Significant *p*-values are indicated in bold. Abbreviations: RLT = Radioligand therapy; VAT = Visceral Adipose Tissue; PSA = Prostate specific antigen

### Overall survival

**Fig. 2:**
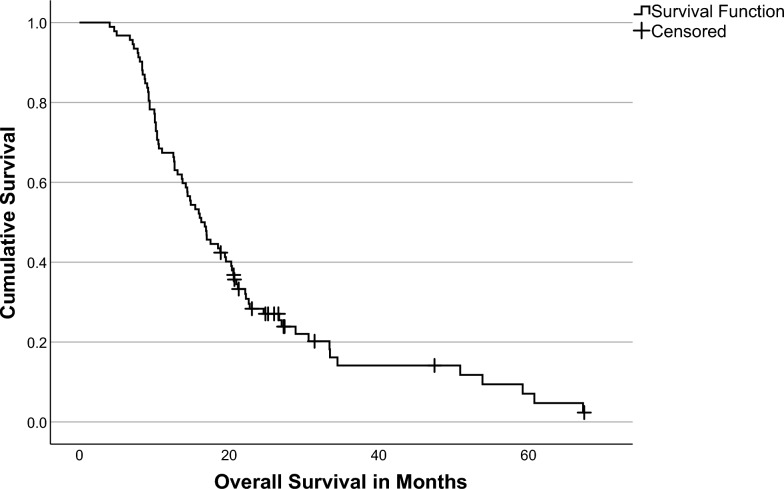
Kaplan–Meier curve of overall survival in the total study population

During follow-up, 78 patients (84.8%) died. Median OS in the total study population was 16.3 months (IQR: 10.1–27.0 months). The median follow-up time in survivors was 25.6 months (IQR: 21.1–28.4 months). The 1 year mortality rate was 32.6% (95%CI 23.0–42.2%) and the 5 year mortality rate was 92.9% (95%CI 85.8–100.0%). Figure 2 shows the Kaplan–Meier curve of OS for the total study population.

### Body composition parameters

Baseline BC parameters and their absolute and relative changes are presented in Table 2.

**Table 2:** Baseline BC parameters with their absolute and relative changes

Body composition parameter	Median (Range)		*p*
	Baseline staging	Interim staging	
BMI	25.9 (15.9–42.9)	25.3 (15.2–44.6)	** < 0.001**
PMI	4.9 (2.7–10.4)	5.1 (2.5–10.1)	0.55
SMI	44.3 (28.3–65.6)	44.4 (26.4–67.1)	0.22
VAT	178.6 (7.0–410.0)	159.8 (2.7–385.4)	**0.033**
SAT	187.2 (10.9–523.6)	176.0 (0.0–525.5)	** < 0.001**

The values at baseline staging and at interim staging were compared using a two-tailed Wilcoxon signed-rank test. Significant *p*-values are indicated in bold. Abbreviations: BMI = Body mass index; PMI = Psoas muscle index; SMI = Skeletal muscle index; VAT = Visceral adipose tissue; SAT = Subcutaneous adipose tissue

The median BMI at the time of baseline staging was 25.9 and slightly decreased to 25.3 at interim staging. This change was statistically significant (*p* = 0.001). Likewise, median VAT decreased significantly from 178.6 to 159.8 between baseline staging and interim staging (*p* = 0.027) as did median SAT from 187.2 to 176.0 (*p* < 0.001). PMI and SMI remained stable and showed no significant alteration between the two examinations.

### Prognostic factors

In univariable Cox regression, presence of lymph node (HR: 0.54) as well as liver metastases (HR: 2.63), previous treatment with Ra-223 (HR: 2.39), previous chemotherapy of any type (HR: 2.50), baseline PSA level (HR: 1.00), hemoglobin level (HR: 0.86), and De Ritis ratio (HR: 1.54) were significant predictors of OS. Additionally, relative changes in SMI (HR: 0.004), relative changes in VAT (HR: 0.34) and changes in SAT (HR: 0.08) were significant prognostic factors of OS. Relative changes in BMI achieved a *p* < 0.1. Baseline BC parameters showed no prognostic value in univariable Cox regression.

In multivariable Cox regression, only the presence of liver metastases (HR: 2.42; 95% CI 1.16–5.03; *p* = 0.006), previous chemotherapy of any type (HR: 2.43; 95% CI 1.37–4.31; *p* = 0.003), baseline De Ritis ratio (HR: 1.40; 95% CI 1.13–1.72; *p* = 0.002), and a relative decrease in VAT (HR: 0.26; 95% CI 0.10–0.67; *p* = 0.006) retained their significant prognostic value. The detailed results of univariable and multivariable Cox regression are presented in Table 3.

**Table 3:** Univariable and multivariable Cox regression

	Univariable Cox regression			Multivariable Cox regression		
Variable	Hazard ratio	95% Confidence interval	*p*-Value	Hazard ratio	95% confidence interval	*p*-Value
Age	1.00	0.97–1.02	0.85			
Height	1.78	0.04–77.75	0.76			
Initial Gleason score	0.99	0.82–1.19	0.88			
*Localization of metastases*						
Lymph node	0.54	0.30–0.95	**0.033**			0.25
Bone	1.79	0.65–4.91	0.26			
Liver	2.63	1.30–5.33	**0.007**	2.42	1.16–5.03	**0.018**
Lungs	1.19	0.64–2.22	0.58			
Soft tissue	1.21	0.58–2.53	0.62			
Adrenal gland	0.64	0.09–4.63	0.66			
*Baseline body composition parameters*						
BMI	0.97	0.91–1.04	0.41			
PMI	0.98	0.84–1.14	0.77			
SMI	0.99	0.96–1.03	0.73			
VAT	1.00	1.00–1.00	0.26			
SAT	1.00	1.00–1.00	0.89			
*Relative changes in body composition parameters*						
BMI	0.01	0.00–1.16	0.057			0.83
PMI	0.86	0.21–3.46	0.83			
SMI	0.004	0.00–0.22	**0.007**			0.23
VAT	0.34	0.13–0.90	**0.03**	0.26	0.10–0.67	**0.006**
SAT	0.08	0.02–0.31	** < 0.001**			0.33
*Pretreatment*						
Radical prostatectomy	0.96	0.61–1.50	0.86			
Androgen deprivation therapy	1.43	0.35–5.91	0.62			
Abiraterone	1.28	0.76–2.17	0.35			
Enzalutamide	1.22	0.76–1.95	0.41			
Radiation therapy	0.85	0.53–1.37	0.52			
Ra-223 therapy	2.39	1.02–5.60	**0.046**			0.15
Chemotherapy of any type (yes vs. no)	2.50	1.45–4.30	** < 0.001**	2.43	1.37–4.31	**0.003**
*Pretherapeutic laboratory values*						
PSA [ng/ml]	1.00	1.000–1.001	** < 0.001**			0.80
Creatinine [mg/dl]	1.78	0.70–4.49	0.23			
Urea [mg/dl]	1.01	0.99–1.03	0.40			
Uric acid [mg/dl]	1.07	0.89–1.28	0.48			
Leukocytes [/nl]	1.07	0.94–1.21	0.31			
Thrombocytes [/nl]	1.00	1.00–1.01	0.18			
Hemoglobin [g/dl]	0.86	0.75–0.98	**0.021**			0.76
De Ritis ratio	1.54	1.27–1.87	** < 0.001**	1.40	1.13–1.72	** < 0.001**

Significant *p*-values are indicated in bold. Abbreviations: BMI = Body mass index; PMI = Psoas muscle index; SMI = Skeletal muscle index; VAT = Visceral adipose tissue; SAT = Subcutaneous adipose tissue; PSA = Prostate specific antigen

**Fig. 3:**
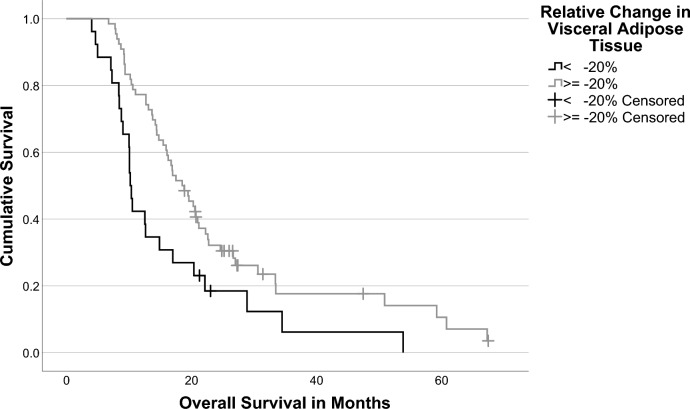
Kaplan–Meier curves of overall survival separated by the relative change in visceral adipose tissue (VAT)

The Charité cutoff finder identified a value of −20% for the relative change in VAT to be the optimum cutoff value. Patients with a high decrease in VAT (< −20%) had a median OS of 10.2 months (IQR: 8.4–20.4 months), while patients with a lower VAT decrease or increase in VAT (≥ −20%) showed a median OS of 18.5 months (IQR: 12.7–30.6 months). This difference was also significant when compared using the log-rank test (*p* = 0.008; Fig. 3).

### Relative change in VAT and best PSA response

In a separate Cox model, the VAT decrease predicted OS (*p* = 0.005) independent of the best PSA response after 1 or 2 cycles of RLT (*p* = 0.09), and there was no significant interaction between the two (*p* = 0.09). Results of the separate Cox model are presented in Table 4.

**Table 4:** Results of multivariable Cox regression including relative change in VAT, best PSA response after 1 or 2 cycles of RLT, and their interaction term

	Multivariable Cox regression		
Variable	Hazard ratio	95% Confidence interval	*p*-Value
Relative change in VAT	0.19	0.06–0.60	**0.005**
Best PSA response	1.08	0.99–1.18	0.09
Relative change in VAT * Best PSA response	0.26	0.06–1.21	0.09

Significant *p*-values are highlighted in bold. Abbreviations: VAT = Visceral adipose tissue; PSA = Prostate specific antigen

### Body composition parameters and relative change in VAT

After subdividing the total study population by high decrease in VAT (< −20%) versus low decrease or increase in VAT (≥ −20%), SAT was significantly lower after the second cycle of RLT in the subgroup with a high decrease in VAT (*p* = 0.008; Table 5). All other BC parameters showed no significant difference.

**Table 5:** Body composition parameters for subgroups separated by the relative change in VAT

Parameter	Median (Range)		*p*
	Baseline staging		
	Relative change in VAT < −20%	Relative change in VAT ≥ −20%	
BMI	25.8 (15.9–32.3)	26.0 (18.7–42.9)	0.53
PMI	4.8 (2.7–10.4)	4.9 (2.7–8.9)	0.17
SMI	44.5 (29.2–61.7)	44.1 (28.3–65.6)	0.94
SAT	170.0 (10.9–315.0)	187.9 (74.5–523.6)	0.26

	Interim staging		
	Relative change in VAT < −20%	Relative change in VAT ≥ −20%	
BMI	24.9 (15.2–31.2)	25.4 (18.7–44.6)	0.14
PMI	4.6 (2.5–10.1)	5.3 (2.8–8.9)	0.08
SMI	43.5 (26.4–59.2)	44.6 (27.9–67.1)	0.40
SAT	146.0 (0.0–254.9)	187.6 (70.8–525.5)	**0.008**

	Relative change		
	Relative change in VAT < −20%	Relative change in VAT ≥ −20%	
BMI	−4% (−13– + 16%)	0% (−20– + 15%	** < 0.001**
PMI	−3% (−42– + 63%)	0% (−43– + 54%)	0.986
SMI	−2% (−23– + 9%)	+ 6% (−15– + 16%)	0.12
SAT	−14% (−100– + 28%)	−1% (−33– + 39%)	** < 0.001**

Body composition parameters are provided separately for patients with a high decrease in VAT (< −20%) versus those with a low decrease or increase in VAT (≥ −20%). The two subgroups were compared using a two-tailed Wilcoxon rank-sum test. Significant p-values are indicated in bold. Abbreviations: BMI = Body mass index; PMI = Psoas muscle index; SMI = Skeletal muscle index; VAT = Visceral adipose tissue; SAT = Subcutaneous adipose tissue

## Discussion

The aim of this analysis was to investigate the prognostic value of monitoring intratherapeutic changes in BC parameters in patients with mCRPC receiving RLT with [^177^Lu]Lu-PSMA. To the best of our knowledge, no such analysis has been conducted before. Our results demonstrate that a high decrease in the VAT during the first two RLT cycle is a significant predictor of shorter survival in multivariable Cox regression analysis. No other BC parameters emerged as relevant predictors of survival.

Recently, Hartrampf et al*.* also investigated the prognostic value of the baseline BMI and CT-based BC parameters for OS after RLT in 171 patients with prostate cancer [21]. Like our analysis, they did not observe significant prognostic value of baseline CT-based BC parameters. This observation is in line with the results from Stangl-Kremser et al*.* who did not find a significant prognostic value of BC parameters (BMI; muscle and fat volumes/indices; visceral-to-subcutaneous fat area ratio; visceral-fat-to-muscle ratio) in patients undergoing first-line treatment for castration-resistant prostate cancer [26]. However, unlike our analysis, Hartrampf et al*.* found the baseline BMI to be a significant prognostic factor for OS after RLT [21]. Differences between the two analyses may arise because Hartrampf et al*.* segmented the tissue types manually, whereas we chose a fully automated AI-based approach. The latter is very time efficient, as the measurement only takes a few seconds, and it ensures good reproducibility [24]. The study by Hartrampf et al*.* also differs in the choice of therapeutic drug, as the authors only used [^177^Lu]Lu-PSMA-I&T, whereas we treated patients with either [^177^Lu]Lu-PSMA-I&T or [^177^Lu]Lu-PSMA-617 [21]. Nevertheless, a recently published matched-pair analysis did not reveal a significant difference in OS between the two substances [27].

In recent years, the peritherapeutic change in VAT has already been identified as an independent prognostic factor of OS and other survival outcomes in various types of cancers [28–30]. For example, in patients with colorectal cancer who underwent curative colectomy, Choe et al*.* showed that patients with a posttherapeutic increase in VAT relative to the baseline value had a significantly longer OS (HR: 0.557) [29]. Furthermore, Zhang et al*.* demonstrated that a reduction in VAT ≥ −35.7% during neoadjuvant treatment was also a significant prognostic factor for both shorter OS and disease-free survival after consecutive gastrectomy in patients with gastric cancer [28]. These findings indicate that in other malignancies VAT could be a valuable on-treatment prognostic factor for the risk of relapse or progression and thus OS.

The pathophysiological mechanisms underlying the association between a stronger decline in VAT and shorter OS remain unclear. Patients with both high and low VAT decline showed no differences regarding BC at baseline and thus had the same initial conditions. One hypothesis is that a drop in VAT could be a sign of developing tumor cachexia, which is reflected in a continuous decrease in skeletal muscle mass and adipose tissue and which is commonly associated with poorer survival [31, 32]. This hypothesis is also supported by the observation that patients with a high decline in VAT compared to those with a smaller decline or increase also had a significantly more pronounced decrease in BMI and SAT between baseline and interim staging, overall implying a catabolic metabolism in these patients. Furthermore, in our separate analysis, there was no interaction between the relative change in VAT and the best PSA response, so it could not be demonstrated that a decrease in VAT is a consequence of biochemical progression of the disease. Side effects of treatment, such as dry mouth, fatigue, nausea, and diarrhea, could potentially exacerbate this pathomechanism by further reducing physical activity and reducing nutrient intake [33]. However, it is not known whether SMI, BMI, VAT, and SAT can be influenced by supportive measures such as nutritional support in critically ill patients with mCRPC and whether such measures will be beneficial for prognosis. Furthermore, patients with (cancer-related) cachexia are also at higher risk of longer hospital stays and increased perioperative complications. The muscle loss associated with these conditions leads to diminished physical function and greater frailty, which complicates recovery and increases susceptibility to infections and other complications [17, 20, 34]. Extended hospital stays and higher complication rates contribute to increased healthcare costs. Therefore, future studies should additionally investigate the influence of a high-calorie diet during RLT, as Hartrampf et al. have already called for [21].

Currently, in addition to rPFS and OS, the blood-based PSA value is used to assess the effectiveness of therapies against metastatic prostate cancer by determining the, so called, PSA response rate [35]. The PSA trend as well as a PSA decline ≥ 30% and ≥ 50% after 1 or 2 cycles of RLT has shown a significant and independent prognostic value for OS in numerous studies [36–38]. Our current analysis shows that the relative change in VAT has a prognostic value that is independent of the best PSA response after 1–2 cycles of RLT and thus might provide additional prognostic information in clinical decision-making.

Besides the change in VAT, our multivariable Cox regression analysis also tested prior chemotherapy of any type, the presence of liver metastases and the pretherapeutic De Ritis ratio as significant and independent prognostic factors for OS. The pretherapeutic De Ritis ratio was already described as an independent prognostic factor of OS in patients with mCRPC undergoing RLT [8]. In addition, chemotherapy-pretreatment and the presence of liver metastases have already been shown in other studies to be significant predictors of a shorter OS [39, 40].

Limitations of this study are its retrospective and single-center design and the lack of a matched control group with different treatments. Therefore, a predictive capability of intratherapeutic alterations in the VAT could not be formally assessed. Prospective studies are required to validate the current explorative results and to ensure a well-selected, homogenous patient population. MTV and TLP were not included in this study as they are not routinely collected at our center. Among patients with a greater decrease in VAT, liver metastases were significantly more frequent, pretherapeutic hemoglobin levels were significantly lower, and they received significantly less Enzalutamide prior to RLT. This could indicate a selection bias. Nevertheless, multivariable Cox regression confirmed the independent prognostic value of the change in VAT even after adjustment for these parameters.

## Conclusions

The prognostic relevance of the VAT trend suggests that more research is needed to determine whether measures to improve VAT, for example through high-calorie food or physiotherapy, can help improve overall patient outcomes. Because changes in VAT emerged as an independent predictor, it is essential to evaluate patients during treatment rather than relying solely on baseline assessments. This is particularly relevant given that mCRPC patients receiving RLT typically undergo interim-staging after two to three of six therapy cycles, allowing for timely monitoring of VAT changes. Moreover, a PACS-integrated, AI-based approach to body composition monitoring is highly time-efficient and cost-effective and it can be easily incorporated into daily clinical practice.

## Data Availability

The datasets used and/or analysed during the current study are available from the corresponding author on reasonable request.

## Abbreviations

mCRPC: Metastatic castration-resistant prostate cancer
OS: Overall survival
RLT: Radioligand therapy
SUVmax: Maximum standardized uptake value
SUVmean: Mean standardized uptake value
TLP: Total-lesion PSMA
MTV: Molecular-tumor volume
PET/CT: Positron emission tomography/computed tomography
BC: Body composition
BMI: Body mass index
AI: Artificial intelligence
SMI: Skeletal muscle index
PMI: Psoas muscle index
VAT: Visceral adipose tissue
SAT: Subcutaneous adipose tissue
PACS: Picture archiving and communications system
L3: Third lumbar vertebra
IQR: Interquartile range
ADT: Androgen deprivation therapy
PSA: Prostate-specific antigen
HR: Hazard ratio
CI: Confidence interval
Ra-223: Radium-223
